# Referencing the sulcus line of the trochlear groove and removing intraoperative parallax errors improve femoral component rotation in total knee arthroplasty

**DOI:** 10.1007/s00167-015-3668-7

**Published:** 2015-06-07

**Authors:** Simon Talbot, Pandelis Dimitriou, Michael Mullen, John Bartlett

**Affiliations:** 1 Western Health, Melbourne, VIC 3011 Australia; 2 Western Infirmary, Glasgow, Scotland; 3 Warringal Private Hospital, Melbourne, Australia

**Keywords:** Knee, Arthroplasty, Total knee arthroplasty, Femur, Rotation, Femoral rotation, Malalignment, Sulcus line, Whiteside’s line, Epicondylar axis, Posterior condylar axis, Trochlear

## Abstract

**Purpose:**

Firstly, to assess and compare the accuracy and reproducibility of the sulcus line compared to Whiteside’s line. Secondly, to assess the accuracy of intraoperative techniques for using the rotational alignment of the trochlear groove to set femoral rotation. Thirdly, to assess the reproducibility of a trochlear alignment guide which removes parallax errors that occur when projecting the sulcus line onto the surface of the femur. Finally, to measure the result of combining the geometrically accurate sulcus line and the posterior condylar axis.

**Methods:**

Three surgeons measured eight rotational angles on ten cadaveric femora. This included Whiteside’s line, the sulcus line and the techniques in which they can be referenced during surgery.

**Results:**

Relative to the anatomical epicondylar axis, the sulcus line (mean −2.8°, SD 2.0°, range −5.4° to 0.8°) had significantly lower variance (*F* = 5.16, *p* = 0.036) than Whiteside’s line (mean −2.0°, SD 3.7°, range −6.0° to 3.4°). The trochlear alignment guide produced the best results of the intraoperative techniques by maintaining the accuracy of the sulcus line and projecting it onto the distal cut surface of the femur without change in rotational angle.

**Conclusion:**

The sulcus line is more accurate and reproducible than Whiteside’s line. Removing parallax errors during surgery improves femoral component rotation. The trochlear alignment guide produced accurate results suggesting that it may be beneficial in a clinical setting. Averaging the sulcus line and posterior condylar axis on the cut surface of the femur improved accuracy over the individual landmarks. Femoral component malrotation is a common cause of patient dissatisfaction and revision surgery. By isolating the rotational alignment of the trochlear groove using the sulcus line, and maintaining its accuracy with an intraoperative guide, we can decrease the risk of femoral component malrotation and improve patient outcomes.

## Introduction

**Fig. 1:**
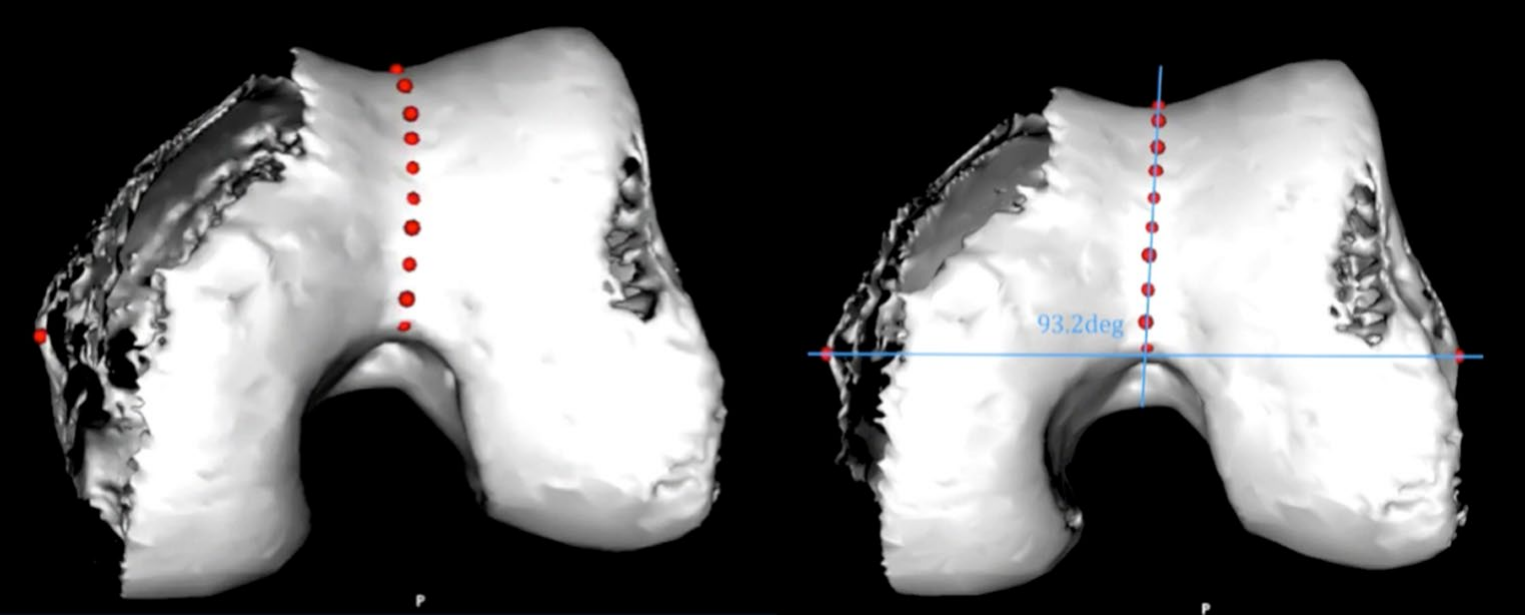
Sulcus line of the trochlear groove allows the rotational alignment of the trochlear groove to be isolated. When the SL is a *straight line*, the surgeon is looking along the coronal alignment of the trochlear groove. In any other coronal orientation, the SL appears as a *curve*

**Fig. 2:**
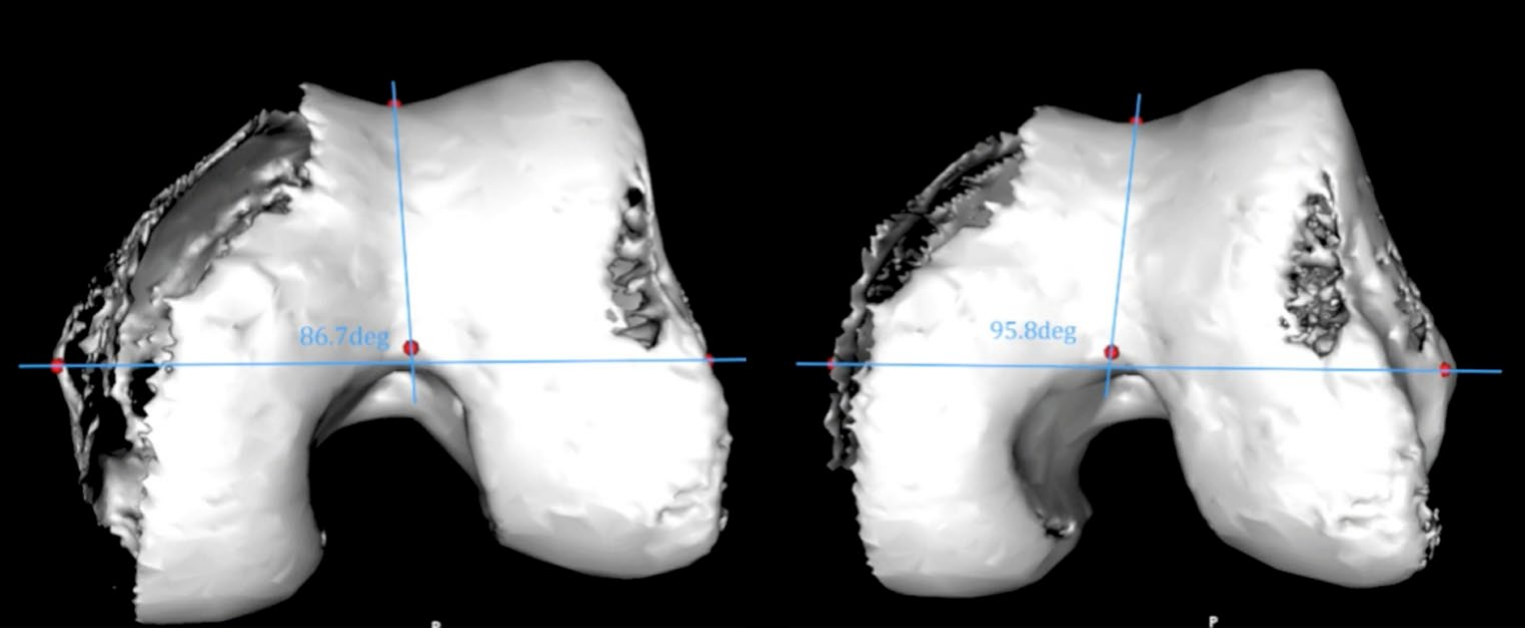
Angle between Whiteside’s line and the fixed epicondylar axis changes depending on the coronal direction in which an observer is looking at the end of the bone. In this case, it changes from 86.7° with a varus viewpoint to 95.8° with a valgus viewpoint. This difference is purely caused by parallax error

Femoral component malrotation is a cause of pain, stiffness, patellofemoral complications and component failure in total knee arthroplasty (TKA) [1, 2, 4, 5, 9, 10, 22, 23]. Current recommendations suggest that the combination of two or more anatomical landmarks or the use of preoperative CT scans may be necessary to improve accuracy [19, 25, 28]. Recent research has described the benefits of the use of the sulcus line (SL) (Fig. 1) over the traditional anteroposterior axis, also known as Whiteside’s line (WL) (Fig. 2) [27]. The SL allows the rotational alignment of the trochlear groove to be more accurately isolated than previous techniques. By maintaining this accuracy with an intraoperative alignment guide, there is a potential to decrease the risk of femoral component malrotation.

The SL uses multiple points along the floor of the trochlear groove. WL only uses two points and one of them is the anterior point in the proximal section of the trochlear groove which has been shown to be inaccurate due to anatomical variation and arthritis [6, 29].

**Fig. 3:**
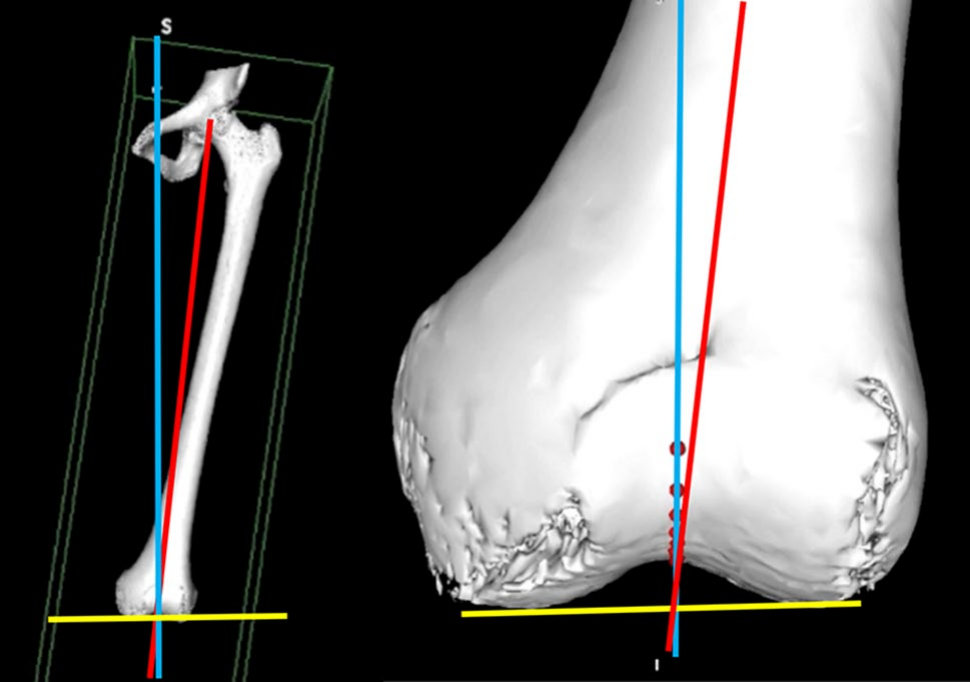
Coronal axis of the sulcus line (CAxSL, *blue line*) often varies widely from the mechanical axis (MAx, *red line*) and the distal condylar axis (DCA, *yellow line*). In this case, the CAxSL is 5° valgus to the MAx. The mean position is 2.9° varus

**Fig. 4:**
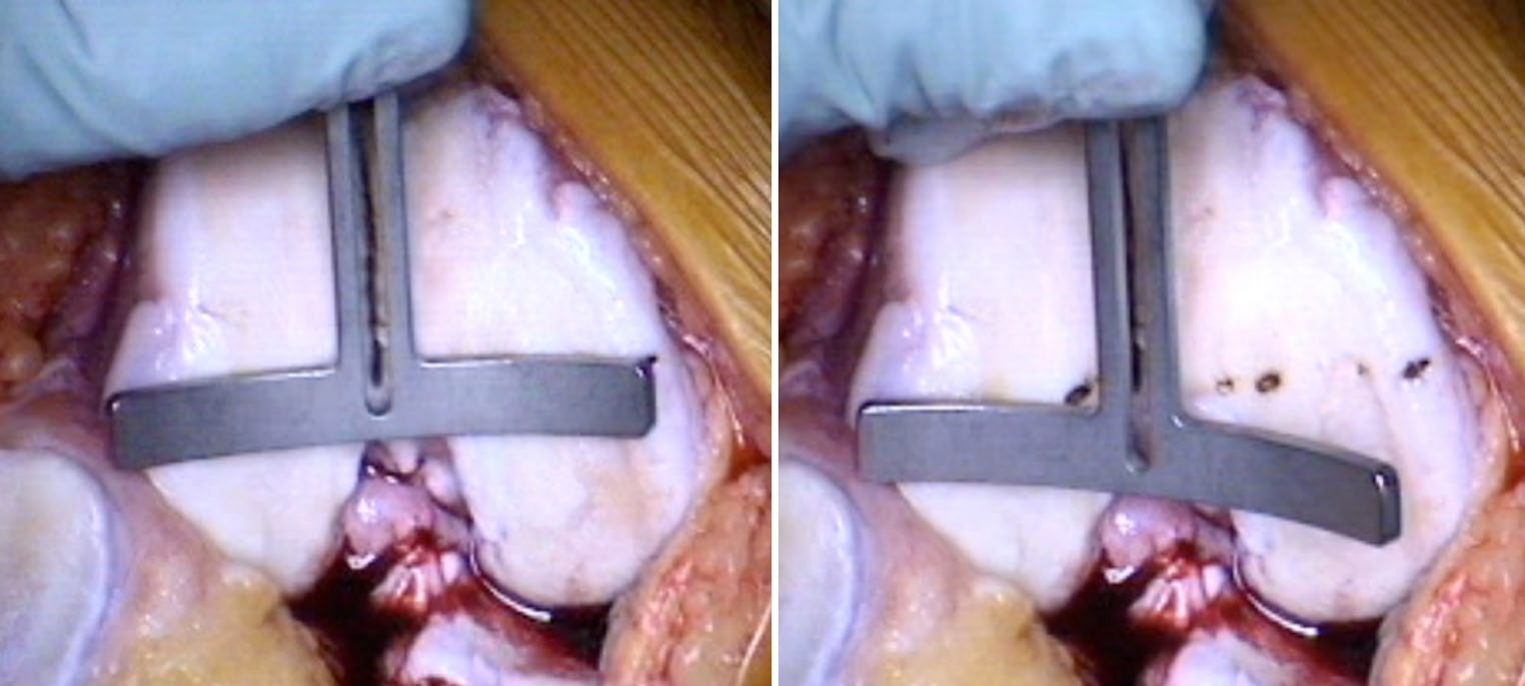
Vertical limb remains aligned with the sulcus line (SL), but the *horizontal limb* does not remain perpendicular as the coronal angle changes. Variations in the *horizontal* (rotational) alignment occur due to a combination of changes in the coronal and sagittal planes. In this demonstration, the error occurs due to variations relative to the position of the fixed camera, whereas during surgery, the variation is due to the difference between the CAxSL and DCA

The collection of multiple points in the trochlear groove also allows the determination of the coronal axis of the sulcus line (CAxSL) (Fig. 3). When viewed along the coronal alignment of the trochlear groove, the SL becomes a straight line rather than a curve. This is the only coronal viewpoint at which the rotational alignment of the trochlear groove can be isolated. Because the coronal alignment of the groove changes in every knee, the SL needs to be measured along a different coronal axis (CAxSL) in every case. Importantly, the CAxSL varies widely from the mechanical axis (MAx) (Fig. 3). This means that the rotational alignment of the trochlear groove cannot be reliably measured along the MAx. If this is attempted, a parallax error occurs whenever the CAxSL and the MAx diverge (Fig. 4).

Because WL is drawn from just two points, there is no way to determine the coronal orientation of the trochlear groove. Because the trochlear groove is often aligned along a different coronal axis, a previously unrecognised parallax error will occur. The size of this error relative to a fixed landmark such as the epicondylar axis can be seen in Fig. 2. This error explains a large portion of the variability which has been described in studies measuring WL [14–16, 18, 21, 27].

By measuring the angle of the SL along the coronal alignment of the trochlear groove, the rotational alignment of the trochlear has been shown to be a more accurate landmark than WL [27]. However, in order to use this landmark during surgery, it needs to be transferred on to the surface of the femur. By transferring the SL onto a surface which is in a different coronal plane, a further parallax error occurs. This surface will be either in the plane of the distal femoral condyles [the distal condylar axis (DCA)] or the cut surface of the femur after the initial distal femoral saw cut (the MAx).

The hypotheses being investigated are, firstly, that parallax errors associated with WL make the SL a more accurate rotational landmark, and secondly, that additional parallax errors occur during surgery as we project the rotational alignment of the trochlear groove onto the femur in order to set femoral component rotation. The aims of this study are to (1) measure the difference in variability between the SL and WL, (2) demonstrate the size of the parallax errors which occur using surgical techniques which reference the trochlear groove to set femoral component rotation, (3) assess the reliability and accuracy of a device designed to remove these errors and transfer the SL on to the distal cut surface of the femur and (4) assess the results of combining the geometrically accurate SL with the posterior condylar axis (PCA).

## Materials and methods

The trochlear alignment guide is a device that corrects parallax errors which occur with current techniques for projecting the rotational alignment of the trochlear groove onto the femur. It does this by matching both the axial rotation and the coronal alignment of the sulcus line of the trochlear groove, and also matching the sagittal alignment of the planned distal femoral cut.

**Fig. 5:**
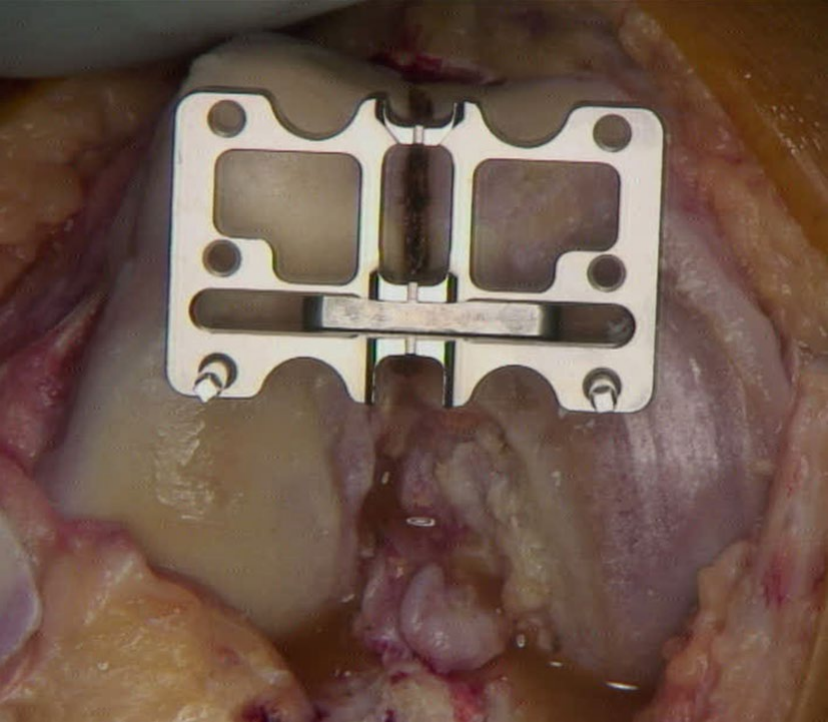
Alignment block can be rotated to match the SL. The block sits over the flattened end of the IM rod. The pinhole tracts will therefore be perpendicular to the planned distal femoral cut in the sagittal plane

**Fig. 6:**
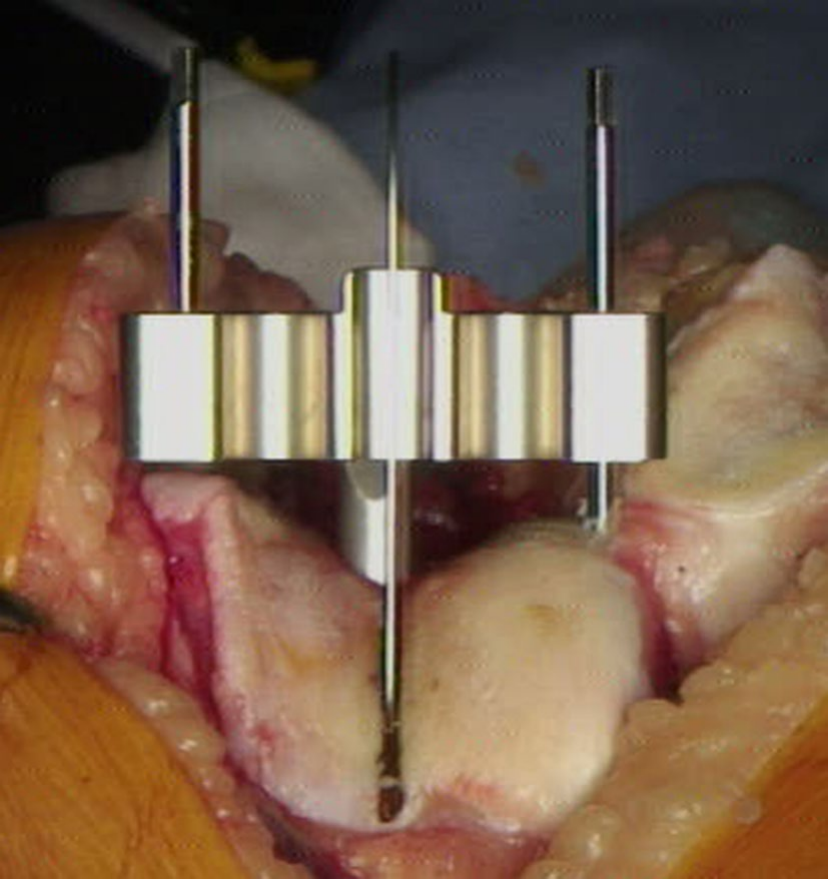
Trochlear alignment guide block matches the coronal alignment of the SL. This is confirmed with the alignment wing. It will usually be in a different coronal plane to the mechanical axis and the distal condyles. In this case, it happens to run along the anatomical axis of the femur, indicated by the IM rod. The trochlear alignment guide is not a cutting block. After it is removed, the standard distal femoral cut can be made using a separate IM rod

The trochlear alignment guide was designed by the senior author (ST) and produced by Allegra Orthopaedics, Sydney, Australia. It consists of four parts: (1) intramedullary (IM) rod, (2) alignment block, (3) alignment wing and (4) two pins. The IM rod has a flattened end which slots into the alignment block. The IM rod ensures that the sagittal plane matches the planned distal femoral cut. The block has a central vertical slot to allow visualisation of the SL. The block can be moved medially and laterally on the flattened end of the IM rod to allow alignment with the SL. The block and rod can together be rotated inside the femoral canal to match the rotation of the SL (Fig. 5). An alignment wing is slotted into the vertical slot in the block to confirm good alignment of the block with both the vertical SL, in the axial plane, and the coronal alignment of the SL when looking from above, in the coronal plane. This often leaves one side of the block sitting off a condyle due to the difference between the coronal alignment of the sulcus line (CAxSL) and the axis across the distal condyles (DCA) (Figs. 2, 6).

The block is held in place with two pins that are inserted through pinholes which are parallel to the IM rod in the sagittal plane, but not necessarily the coronal plane. The trochlear alignment guide does not influence the coronal alignment of the planned distal femoral cut. The trochlear alignment guide, including the pins, is then completely removed. Once the distal femoral cut is made, using a separate IM rod and standard instruments, the pin-tracks from the trochlear alignment guide are seen on the distal femoral cut surface. As the pin-tracks are perpendicular to the distal femoral cut, in the sagittal plane, the rotational orientation of the SL is accurately transferred onto the cut surface.

### Cadaver study procedure

The soft tissues were largely removed from ten embalmed cadaveric femora, apart from the bony attachment of the ligaments and the articular cartilage. Seven of the femora showed no signs of osteoarthritis and three had moderate medial compartment osteoarthritis.

The femora were placed in a multi-axial vice. Pins were placed into the centre of the femoral head in two orthogonal planes and into the medial and lateral epicondyles by a single surgeon (MM). These were used to align the camera to the appropriate axes of the femur and to allow measurement of the various landmarks in comparison with the anatomical epicondylar axis (AEA). To improve accuracy, the medial pin was inserted into the epicondyle to avoid difficulty finding the medial sulcus on any of the specimens.

One orthopaedic surgeon specialising in knee surgery, one orthopaedic fellow and one registrar (ST, MM and PD) participated in the study.

Measurements were recorded by taking a standardised digital photograph. The camera was aligned with the mechanical axis (MAx) or coronal axis of the sulcus line (CAxSL) prior to each picture being taken. The MAx was determined by aligning the orthogonal pins through the femoral head with the centre of the knee joint. The CAxSL was determined by altering the coronal viewpoint of the camera until the curve of the SL became a straight line and the camera was looking along the length of the trochlear groove.

The following eight measurements were taken: (1) vertical SL measured along the CAxSL (90° subtracted to allow comparison with horizontal landmarks), (2) the WL measured between the most proximal and most posterior points marked along the SL, measured along the MAx (90° subtracted), (3) T-piece along the MAx, (4) navigation stylus measured along the MAx, (5) trochlear alignment guide held unpinned along the MAx, (6) the pin-holes created by pinning the trochlear alignment guide on the surface of the femoral condyles, measured along the MAx, (7) the pin-holes created by pinning the trochlear alignment guide on the distal cut surface of the femur, measured along the MAx and (8) the PCA. Techniques 1–5 were performed twice, on each specimen, by all participating surgeons. Techniques 6–8 were performed by only the senior surgeon as it is difficult to blind additional surgeons to the pinholes. All measurements are taken looking along the MAx, except the initial measurement of the SL which is made looking along the CAxSL.

The SL was drawn by the senior surgeon with a permanent marker pen. The trochlear groove was palpated with a thumb, and multiple points were marked in the groove in the manner previously described [26, 27]. The most proximal section of the trochlear groove was ignored if it diverged from the more distal, vertical section. This line was first measured using the alignment wing along the CAxSL. The remaining techniques were then measured with the camera aligned along the MAx. The T-piece was held along the SL, followed by a navigation stylus.

**Fig. 7:**
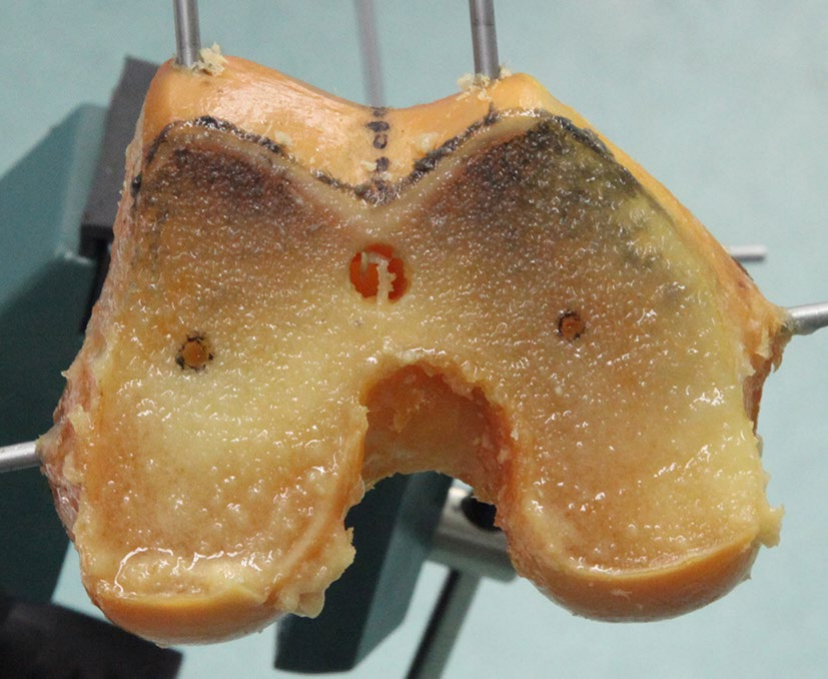
Pin-holes in cut surface of distal femur. This femur demonstrated the most internally rotated measurement of 5.8° between the pin-holes and the AEA

A drill was used to open the intramedullary canal through the centre of the knee. The IM rod was inserted and the trochlear alignment guide applied. The block was aligned and held in place, whilst the photographs were taken along the MAx. The block was then definitively pinned by the senior surgeon and the entire device and pins removed. The position of the pin-holes on the distal condylar surface was photographed. A standard distal femoral cut was made using an IM rod, a 6° valgus cutting block and a 10-mm resection. The pin-holes were identified on the cut surface and photographed (Fig. 7). The PCA was measured on the final photograph. A further axis was calculated by averaging the SL, represented by the pin-holes on the distal femoral cut surface, and the PCA + 3°.

The CAxSL and the DCA were measured relative to the MAx using a goniometer with the posterior condyles resting on a flat surface. Varus and valgus were assigned positive and negative values, respectively.

All photographs were imported into Adobe Photoshop CS6 software. Two independent assessors measured the angles between the measured axis and the centre of the entry points of the epicondylar pins.

Approval was obtained from the Department of Anatomy and Neuroscience of the University of Melbourne under Human Research Ethics Committee approval No. 0608479/2007.

### Statistical analysis

Analyses to determine intraclass correlation coefficients, means, standard deviation (SD) and ranges were conducted using the Statistical Package for the Social Sciences (SPSS) v16.0. To compare variance and means independent samples, *T* tests were conducted.

## Results

All measurements are relative to the anatomical epicondylar axis marked by the pins (AEA). The results of the rotational measurements are summarised in Table 1.

**Table 1:** Rotational measurements

	Mean	SD	Range
SL with wing (measured along CAxSL)	−2.8°^b^	2.0°^a^	−5.4° to 0.8°
WL (APA)	−2.0°	3.7°^a^	−6.0° to 3.4°
T-piece	−2.0°	3.1°	−8.3° to 3.5°
Navigation stylus	−2.2°	3.3°	−8.3° to 3.9°
TAG unpinned	−2.8°	2.1°	−5.9° to 0.8°
Pinholes on condyles	−2.8°	2.2°	−5.8° to 0.0°
Pinholes on cut surface	−2.8°^b^	2.1°	−5.8° to 0.0°
PCA	−5.0°	2.5°	−9.7° to −1.0°
Average of SL using TAG and PCA + 3°	−2.4°	1.9°	−5.9° to −0.4°

All angles are relative to AEA. Negative numbers are internally rotated

All measurements were taken along the viewpoint of the MAx apart from the SL with the alignment wing, which was measured along the CAxSL

^a^Decreased variance for SL compared to WL (*F* = 5.16, *p* = 0.036)

^b^No difference between SL and Pinholes on cut surface

The SL, measured along its coronal axis, produced a mean of −2.8° (SD 2.0°, range −5.4° to 0.8°). WL measured along the MAx produced a mean of −2.0° (SD 3.7°, range −6.0° to 3.4°). The SL had a significantly lower variance than WL (*F* = 5.16, *p* = 0.036).

In assessing the ability of surgeons to isolate and measure the SL, the Pearson’s coefficients for intraobserver reliability were very high at *r* = 0.78, 0.83 and 0.86, increasing with the experience of the surgeon with the landmark. Interobserver reliability was also high with *r* = 0.87, 0.69 and 0.65.

The mean difference between the T-piece and SL measured along its coronal axis was 1.7° (range 0.1°–5.4°), and between navigation stylus and SL was 2.2° (range 0.8°–6.3°).

In assessing the use of the trochlear alignment guide, the Pearson’s coefficients for intraobserver reliability were high with *r* = 0.66, 0.72 and 0.77, tending to increase with the surgeon’s familiarity with the device. Interobserver reliability was similar at 0.56, 0.65 and 0.81 (Table 2).

**Table 2:** Coronal measurements

	Mean	SD	Range
CAxSL to MAx	2.9°	3.1°	−2° to 7°
DCA to MAx	87.3°	1.9°	84° to 90°
DCA to CAxSL	84.4°	2.8°	81° to 89°

Mean CAxSL 2.9° varus to MAx. DCA measured on lateral side of MAx or CAxSL

When the trochlear alignment guide was finally pinned, the pinholes measured on the distal condylar surface produced a mean of −2.8° (SD 2.2°, range −5.9° to 0.0°) and the pinholes on the distal cut surface produced a mean of −2.8° (SD 2.1°, range −5.8° to 0.0°). Correlation between the two measurements was *r* = 0.99. This indicates that there was no loss of accuracy due to parallax between the two surfaces.

There was no significant difference between the SL and the pinholes on the distal cut surface of the femur produced by the trochlear alignment guide, indicating that the trochlear alignment guide was able to accurately project the rotational alignment of the trochlear groove (SL) onto the distal femur without a change in angle.

## Discussion

The results of this cadaver study confirm the predictions from the previous CT study that the sulcus line is more accurate than WL. It also shows that the accuracy of the sulcus line can be maintained intraoperatively using a simple alignment guide.

The SL is a curve formed from the lowest points along the floor of the trochlear groove. This allows orientation to the coronal alignment of the trochlear groove. This removes parallax error which is inherent in the use of WL. The significant difference in variability between the measurement of our SL along the CAxSL and along the MAx can only be attributed to this parallax error as WL was taken form the most anterior and posterior points of the SL. These results are consistent with the previous CT study which also found a significant decrease in variance and reported a standard deviations for the SL of 2.7° (range −4.9° to +4.7°) and WL measured along the MAx of 4.2° (range −11.8° to +7.8°) [27]. These results suggest that the SL has similar or less variability compared to other techniques which have been assessed with post-operative CT scans relative to the SEA (Table 3).

**Table 3:** Results of SL compared to studies assessing rotation with CT scans relative to the SEA

Authors	Axis	*N*	Mean	SD	Range
Luyckx et al. [13]	Preoperative CT	48	2.4°	2.5°	−2.8° to 6.9°
	Gap-balancing	48	1.7°	2.1°	−2.5° to 6.5°
Stockl et al. [25]	PCA + 3°	32	1.1°	2.8°	−2° to 12°
	APA and epicondylar	32	−0.4°	2.4°	−7° to 4°
Seo et al. [24]	Mechanical axis-derived	120	1.6°	2.2°	−4.8° to 7.9°
Talbot et al. [27]	Sulcus line	44	0.3°	2.7°	−4.9° to 4.7°
	PCA + 3°	44	0.7°	2.5°	−5.7° to 7.1°
Current cadaver study	Sulcus line	10	−2.8° (AEA)	2.0°	−5.4° to 0.8°
	PCA + 3°	10	−2.0° (AEA)	2.5°	−9.7° to −1.0°

Drawing the SL during surgery is technically relatively easy. However, it needs to be appreciated that it is not WL. It is best done by careful palpation leading up from the intercondylar notch. This vertical section is not affected by trochlear dysplasia and patellofemoral osteoarthritis which can obliterate the proximal section of the groove. Several studies have confirmed that the proximal section, which is referenced in WL, is prone to excessive variability [6, 29]. Therefore, the distal section of the trochlear, which largely runs in the appropriate axial plane, is used but the most proximal 1–2 cm of the groove is not referenced.

The increased variability measured with the use of the T-piece and the navigation stylus demonstrates the second type of parallax error. This occurs when aligning either instrument with the vertical sulcus line by looking along the coronal alignment of the groove and then projecting that angle onto a surface which lies in a different coronal plane. It happens when drawing a horizontal line across the distal femoral condyles (DCA) with the T-piece or using a computer navigation system to project the alignment of the stylus onto the mechanical axis. In both cases, the error occurs due to a combination of flexion or extension of the instrument in the sagittal plane with the difference between the CAxSL and the DCA or MAx. The magnitude of these errors can be seen by the difference between the SL measurement and the T-piece [mean error 1.7° (range 0.1°–5.4°)], and stylus measurements [mean error 2.2° (range 0.8°–6.3°)]. With all three measurements, the surgeon was aligning the vertical limb of the instrument with the same vertical SL. The likely size of the parallax error can also be estimated by calculation using the formula tan*θ*_3_ = sin*θ*_1_sin*θ*_2_⁄cos*θ*_1_ where *θ*_1_ = coronal plane variation (CAxSL–DCA), *θ*_2_ = sagittal plane variation of T-piece to planned distal femoral cut and *θ*_3_ = resultant rotational variation in axial plane. Applying this formula, it can be calculated that a 5.6° difference between the CAxSL and the DCA (the mean difference detected in this study, Table 2) coupled with a 20° flexion of the T-piece would produce an average 1.9° rotational error. A retrospective review of a large series of femoral components inserted solely using the SL and the T-piece was published alongside the 3DCT study. It produced similar results to our use of the T-piece in cadavers with a mean −3.2° (SD 2.9°, range −10.8° to 3.2°) relative to the AEA [27].

Another common technique is to perform the distal femoral cut and then orientate the sizing guide or cutting block to match the remaining section of the SL or WL. In this situation, there is no ability to match the coronal alignment of the SL and therefore the results will match those of the less accurate WL, even if a significant portion of the SL is still visible.

Preoperative planning techniques for producing patient-specific guides are also prone to parallax error as they routinely reference the groove using Whiteside’s two point definition and fail to account for the coronal alignment of the groove. These issues could be addressed by isolating the coronal alignment of the groove using the SL and orientating the 3D reconstruction prior to the measurement.

This cadaver study confirms the findings in the CT study that the rotational alignment of the trochlear groove, shown as the SL, is a more accurate and reliable landmark than WL. It was significantly less variable than WL. This reproduces the results, in a cadaver model, of the 3DCT study [27]. It should be noted that these measurements were all taken relative to the anatomical epicondylar axis as it was felt that this could be more reliably identified on the cadavers than the surgical epicondylar axis. The SEA is more likely to approximate the desired flexion–extension axis of the knee [3, 7, 11, 12, 30]. The previous CT study recorded that the AEA was on average 3.7° externally rotated to the SEA [27]. This would indicate that our cadaver SL was approximately 0.9° externally rotated to the SEA.

The trochlear alignment guide was able to transfer the rotational alignment of the SL onto both the distal surface of the femoral condyles and the distal cut surface of the femur without any change in the angle. There was a high degree of interobserver and intraobserver reproducibility. Once the SL has been projected onto the distal femoral cut, it is very easy to accurately compare it to the PCA. The results show that whilst the PCA was more variable than the SL in this group, there was no difference between the means. By averaging the individual measurements for the SL and PCA, the overall variability was reduced even further (SD 1.9°). This parallels the data from the 3DCT study that predicted a considerable decrease in the number of outliers (>3° from SEA) by combining the SL and PCA. Paternostre et al. recently reported on the relationship between the PCA and WL on preoperative patient-specific instrument planning. They found a consistent relationship but noted a high degree of variability between the WL and the SEA (range 88.2°–100.6°). This leads to an increased range (90.4°–103.2°) when the two landmarks were combined [19]. This study and the previous 3DCT study suggest that the SL shows less variability than WL and that accurately combining the SL with PCA leads to less variability than using the individual axes. Several other studies have recommended the combination of rotational landmarks to improve clinical outcomes [8, 17, 25]. Piriou et al. [20] used a computer navigation system to align their TKA with trochlear groove and produced good clinical results.

The limitations of this study include the relatively small numbers and the use of cadaveric femora. Further studies with larger numbers would be required to confirm the clinical importance of the variations and to assess the use of the alignment guide intraoperatively.

The concepts introduced in the previous CT-based study [27] and confirmed in this cadaver study have important implications for the way in which we determine the rotational alignment of our TKA. These concepts can be applied to our current techniques for PSI planning and computer navigation as well as conventional surgery. Referencing the trochlear groove using Whiteside’s definition has been widely shown to be inaccurate [28]. When a different method is adopted, which accounts for the three-dimensional nature of the groove, the trochlear can be a reliable landmark for femoral component rotation.

## Conclusion

In order to preserve the accuracy of the SL during surgery, we need to continue to respect its three-dimensional nature. For the SL to be a useful landmark, it needs to be visible on the distal femoral cut surface so that we can use it to orientate our sizing guide or cutting block. This requires a technique which projects the line from one plane, the CAxSL, onto another plane, the MAx, without the risk of parallax error. All of the current techniques for doing this do not account for this error and therefore we have developed a new trochlear alignment guide. Once the SL is projected accurately onto the cut surface of the femur, it can be compared and combined with other landmarks. The rate of femoral component malrotation can be further reduced by combining the SL and the PCA. This technique is reproducible and very simple to perform. It could be readily adopted by surgeons using conventional instruments and used with any type of knee replacement. The application of theses concepts has the potential to decrease the rate of femoral component malrotation and improve outcomes for patients.
